# Inflammaging and fatty acid oxidation in monocytes and macrophages

**DOI:** 10.1097/IN9.0000000000000038

**Published:** 2024-01-19

**Authors:** Victor Kruglov, In Hwa Jang, Christina D. Camell

**Affiliations:** 1 Department of Biochemistry, Molecular Biology, and Biophysics, Institute on the Biology of Aging and Metabolism, University of Minnesota, Minneapolis, MN, USA

**Keywords:** aging, cytokines, fatty acid oxidation, inflammaging, inflammasome, inflammation, lipid droplets, macrophages, metabolic disease, microglia, monocytes

## Abstract

Fatty acid oxidation (FAO), primarily known as β-oxidation, plays a crucial role in breaking down fatty acids within mitochondria and peroxisomes to produce cellular energy and preventing metabolic dysfunction. Myeloid cells, including macrophages, microglia, and monocytes, rely on FAO to perform essential cellular functions and uphold tissue homeostasis. As individuals age, these cells show signs of inflammaging, a condition that includes a chronic onset of low-grade inflammation and a decline in metabolic function. These lead to changes in fatty acid metabolism and a decline in FAO pathways. Recent studies have shed light on metabolic shifts occurring in macrophages and monocytes during aging, correlating with an altered tissue environment and the onset of inflammaging. This review aims to provide insights into the connection of inflammatory pathways and altered FAO in macrophages and monocytes from older organisms. We describe a model in which there is an extended activation of receptor for advanced glycation end products, nuclear factor-κB (NF-κB) and the nod-like receptor family pyrin domain containing 3 inflammasome within macrophages and monocytes. This leads to an increased level of glycolysis, and also promotes pro-inflammatory cytokine production and signaling. As a result, FAO-related enzymes such as 5′ AMP-activated protein kinase and peroxisome proliferator-activated receptor-α are reduced, adding to the escalation of inflammation, accumulation of lipids, and heightened cellular stress. We examine the existing body of literature focused on changes in FAO signaling within macrophages and monocytes and their contribution to the process of inflammaging.

## 1. Introduction

Inflammaging, a hallmark of an aging immune system, is characterized by a persistent low-grade inflammation that increases the risk for age-related diseases ^[1–3]^. Macrophages and monocytes play a crucial role in identifying cellular stress as stimuli, leading to sustained chronic inflammation. These essential cells encompass bone marrow-derived monocytes, lymphoid organ-residing macrophages, and tissue-specific macrophages like microglia and Kupffer cells. With age, these cells exhibit an enhanced pro-inflammatory state and undergo a well-defined decline in common functions such as phagocytosis and antigen presentation ^[2]^. Recently, there has been greater appreciation for the role of metabolic pathways within macrophages and monocytes in the aging immune system. This review will focus on the literature that investigates the intersection of fatty acid oxidation (FAO) with inflammation in macrophages and monocytes during aging. First, we will briefly review important metabolic pathways and their age-related changes. Then, we will discuss the role of FAO in macrophage polarization. Finally, we will examine the pathways of the nod-like receptor family pyrin domain containing 3 (NLRP3) inflammasome, peroxisome proliferator-activated receptor-α (PPAR-α), and 5′ adenosine monophosphate-activated protein kinase (AMPK), and address how alterations in these pathways with age contribute to dysfunctional FAO and inflammaging.

Historically, macrophages were classified based on their polarization and functional roles in response to inflammatory stimuli. Classically activated macrophages (also called M1 macrophages) play essential roles in host defense and antimicrobial functions, whereas alternatively activated macrophages (M2 macrophages) are primarily involved in immune regulation and tissue repair ^[4]^. M1 macrophages are characterized by activated pathways such as toll-like receptor (TLR), nuclear factor-κB (NF-κB), and mitogen-activated protein kinase (MAPK), and high expression of inducible nitric oxide synthase. Initial research in the field demonstrated that saturated fatty acids can induce polarization of macrophages toward the M1 phenotype. This established a connection between lipid exposure and macrophage inflammation ^[5]^. In contrast with M1 macrophages, STAT6, PPAR-γ and Arginase1 are activated in M2 macrophages ^[6]^. Numerous reviews delve into macrophage biology and cellular metabolism ^[6–9]^. This review aims to focus more specifically on the available literature examining the impact of age on FAO in monocytes and macrophages.

## 2. Metabolic pathways

In healthy individuals, lipid catabolism and anabolism are coordinated across tissues to maintain homeostasis. Triglyceride lipolysis appropriately regulates fatty acid release to meet energy demands. These fatty acids undergo sufficient oxidation for acetyl-CoA utilization in the tricarboxylic acid (TCA) cycle and electron transport chain to generate adenosine triphosphate (ATP). Meanwhile, excess nutrients get stored as newly synthesized fatty acids and triglycerides for later use. Catabolism dominates to meet immediate energy needs, while anabolism replenishes lipid stores. This balance between lipid breakdown, oxidation, synthesis, and storage sustains both lipid and systemic energy homeostasis (see relevant pathways below).

**LIPOLYSIS:** This is the process of breaking down stored fats, or triglycerides, into their constituent elements, which can be utilized for energy in cells. The process occurs primarily in adipose tissue and is regulated by a variety of hormones such as insulin, epinephrine, norepinephrine (NE), and glucagon. NE serves as a hormone generated by the adrenal glands and a neurotransmitter/chemical messenger synthesized to facilitating signal transmission from the sympathetic nervous system. Adipose lipases hydrolyze stored triglycerides that are found in lipid droplets to free fatty acids and glycerol. Free fatty acids are then released into the bloodstream and used as a source of energy, whereas glycerol is used in gluconeogenesis.

**FATTY ACID OXIDATION:** While there are various mechanisms to degrade fatty acids, including α-, β-, and ω-oxidation, the majority of FAO occurs in mitochondrial β-oxidation, where fatty acids are broken down into acetyl-CoA, and subsequently utilized by the TCA cycle. This process generates nicotinamide adenine dinucleotide + hydrogen (NADH) and dihydroflavine-adenine dinucleotide (FADH_2_). Peroxisomal β-oxidation is responsible for the degradation of very long-chain fatty acids, for further catabolism in the mitochondria.

**OXIDATIVE PHOSPHORYLATION:** During this process, electrons from NADH and FADH_2_ (generated from FAO and the TCA cycle) are transferred to the electron transport chain. This generates a proton gradient that is utilized by ATP synthase to catalyze the phosphorylation of adenosine diphosphate (ADP) to produce ATP. This process generates most of the energy in the cell.

**FATTY ACID SYNTHESIS:** In fatty acid synthesis, the initial step involves converting acetyl-CoA into malonyl-CoA. This conversion is facilitated by the rate-limiting enzyme called acetyl-CoA carboxylase (ACC). Subsequently, malonyl-CoA serves as a precursor for the production of long-chain saturated fatty acids. This process is carried out by the enzyme fatty acid synthase. These processes take place within the cell’s cytoplasm and require ATP and reducing equivalents like nicotinamide adenine dinucleotide phosphate + hydrogen (NADPH).

**TRIGLYCERIDE SYNTHESIS:** This process unfolds within the endoplasmic reticulum of cells and requires diacylglycerol acyltransferase (DGAT). This enzyme orchestrates ester bond formation by merging fatty acyl-CoA compounds with the unoccupied hydroxyl groups in diacylglycerols. Comprised of glycerol and three fatty acids, triglycerides constitute a principal reservoir of stored energy. This process is controlled, responding to dietary inputs, hormonal cues (eg, insulin), and the body’s energy demands.

Aging leads to significant alterations in metabolic pathways across various tissues. Adipose tissue exhibits signs of immune activation and metabolic dysregulation prior to their presence in other tissues ^[10–13]^. These alterations lead to age-related weight gain, increased visceral adiposity, and shifts in energy utilization patterns, that increase the risk of cardiovascular disease and metabolic syndrome ^[14,15]^. Cardiac and skeletal muscle also exhibit a decreased FAO efficiency due to impaired mitochondrial function ^[16,17]^. The reduction in FAO along with an increase in fatty acid synthesis, results in ectopic lipid accumulation and lipotoxicity in those tissues ^[18]^. Several key publications provide evidence for a reduction in FAO in monocytes and macrophages during aging ^[19–32]^; these results are supported by literature that shows many cell types exhibit enhanced glycolytic activation, but reduced FAO pathways ^[19,33]^.

## 3. Fatty acid oxidation as a critical metabolic pathway in macrophage polarization

Cellular metabolism is a critical component of macrophage function, for a recent review that covers multiple metabolic pathways, please see Russel et al ^[34]^. Here, we provide a brief overview of macrophage polarization. In vitro macrophages can be stimulated with interferon-gamma (IFN-γ) and lipopolysaccharide (LPS) to induce a classically activated macrophage (M1 macrophage), whereas treatment with interleukin 4 and 13 (IL-4 and IL-13), generates an alternatively activated macrophage (M2 macrophage) ^[35,36]^. Previous research has shown that M2 macrophages exhibit increased energy demand reliant on FAO, oxidative phosphorylation, and glutamine metabolism, but reduced utilization of glycolysis. On the other hand, M1 macrophages display decreased FAO, but increased glycolytic metabolism, nitric oxide production and propensity for nucleotide biosynthesis and reactive oxygen species (ROS) ^[8]^. The activity of these metabolic pathways supports the phenotypic function of polarized macrophages. In particular, robust enhancement of glycolysis supports a break in the TCA cycle in M1 macrophages, increasing the activation of the NLRP3 inflammasome (see Section 4), via the TCA metabolites ^[37–39]^. The production of nitric oxide by murine macrophages is linked to citrate accumulation, which supports lipid synthesis and inflammation ^[40]^. Importantly, M1 macrophages exhibit more lipid droplets that are not seen in M2 macrophages ^[20,41,42]^. These results suggest that M2 macrophages more actively engage in FAO, and increased levels of FAO in M2 macrophages lead to a constant supply of energy, an uninterrupted TCA cycle, increased oxidative phosphorylation, aid in the resolution of inflammation, and the promotion of tissue homeostasis and repair ^[43]^.

A critical research question is: is FAO required for polarization in macrophages? Mitochondrial enzymes, carnitine palmitoyltransferases I and II (CPT1 and CPT2) that transfer fatty acid chains across the mitochondrial membrane, are required for the oxidation of long-chain fatty acids. A prior study found that treatment of M2-polarized macrophages with etomoxir, a CPT1-inhibitor, reduces M2 markers, CD206, resistin-like molecule alpha (RELMα), and CD301 expression ^[44]^. Furthermore, it was shown that etomoxir reduces spare and respiratory capacity and oxygen consumption rate of those M2 macrophages, indicative of lower oxidative phosphorylation. However, additional studies utilizing etomoxir have demonstrated that FAO inhibition prior to macrophage polarization is not sufficient to completely abrogate IL-4-induced M2 polarization ^[45–47]^. Moreover, the inhibitory effects of etomoxir on M2 polarization at high doses also involve CPT1-independent mechanisms, such as the depletion of intracellular levels of coenzyme A ^[48]^. Furthermore, macrophages lacking *Cpt2* still undergo M2 polarization in the presence of IL-4, assessed by M2 markers *Arg1*, *Mgl2*, and *Retnla* ^[49]^. Overall, the current research indicates that FAO is not required for M2 phenotype; rather, FAO supports M2 macrophage function.

TCA intermediates, also contribute to pro-inflammatory and anti-inflammatory characteristics ^[8]^. These molecules modulate inflammation and metabolic function in both autocrine and paracrine manners, and some (eg, citrate or itaconate) directly influence FAO ^[43,50]^. Citrate, itaconate, and succinate are highly upregulated upon M1 macrophage activation, as TCA cycle intermediates accumulate due to a break in the cycle ^[8]^. Accumulated citrate and succinate further promote an inflammatory M1 phenotype. However, itaconate modulates inflammation in a complex manner. It can exhibit anti-inflammatory effects by inhibiting M1 macrophage-mediated inflammation. Specifically, it suppresses interferon signaling pathways by activation of the Nrf2 antioxidant pathway, and the inhibition of succinate dehydrogenase ^[51,52]^. At the same time, it has been shown that itaconate and its derivates suppress M2 macrophage activation through the inhibition of JAK1 phosphorylation through cysteine modifications ^[53]^. To date, these data suggest that TCA intermediates regulate M1 and M2 macrophage polarization.

In vivo, macrophages do not exhibit strict M1 or M2 phenotypes, but instead are found on a spectrum of phenotypes. The relationships between macrophage phenotype and lipid metabolism are linked to stimuli from the local environment. For example, in adipose tissue, stimuli may be from local cell types such as dying adipocytes—that lead to crown-like structures (CLS), or secreted factors such as adiponectin, IL-10, IL-4, and IL-1β ^[54]^. CLS refers to the macrophages that surround those adipocytes and serve as a hallmark of ongoing inflammation and adipocyte cell death. While healthy adipocytes secrete adiponectin and IL-10 which supports the M2 phenotype, CLS-derived macrophages exhibit an inflammatory, lipid-laden M1 phenotype ^[55,56]^. Recent work also indicates that the oxidative phosphorylation pathway and transcriptional signature significantly distinguishes tissue-resident macrophages from each other, depending on their organ location ^[57]^. In this work, myeloid-specific deletion of mitochondrial transcription factor A (TFAM) that controls the transcription of mitochondria-encoded genes causes a reduction of oxidative phosphorylation and apoptosis of those macrophages in multiple tissues ^[57]^. These data support the premise that macrophages in vivo require the oxidative phosphorylation pathway to support their functions. In this review, following the nomenclature of the reviewed literature, we will continue to use M1 or M2 to represent a dominant phenotype of the examined cell.

Macrophage phenotype is also reflected by an abundance of specific lipid species ^[58]^. Morgan et al ^[59]^ quantified lipid profiles in in vitro generated, M1 and M2 macrophages, and in adipose tissue macrophage subsets. M1 macrophages exhibit distinctive lipids, such as higher levels of triacylglycerols and cholesterol esters. Triglycerides found in M1 macrophages are rich in polyunsaturated fatty acids. In contrast, M2 macrophages are characterized by a higher abundance of glycerophospholipids, ether lipids, and sphingolipids. Additionally, the triglycerides in M2 macrophages are predominantly saturated fatty acids. Akin to in vitro polarized macrophages, in vivo M1-like CD9^+^ macrophages that reside in CLS feature polyunsaturated triglycerides, whereas adipogenic M2-like Ly6C^+^ macrophages have triglycerides enriched in saturated and monounsaturated fatty acids. CD9^+^ and Ly6C^+^ macrophages also differ significantly in their sphingolipid content and phospholipid proportions. Thus, the polarization and preferential use of metabolic conditions within macrophage subsets direct exogenous fatty acids toward internal lipid storage in various ways. The ability of M1 macrophages to deal with lipotoxicity is due to the increased activity of DGAT, an enzyme responsible for converting free fatty acids into triglycerides. Diacylglycerol *O*-acyltransferase (*Dgat1*) expression increases in adipose tissue macrophages from mice fed a high-fat diet ^[60]^. Macrophage-specific overexpression of DGAT in mice are protected from diet-induced insulin resistance and inflammation, by turning free fatty acids into neutral lipids that can be stored in lipid droplets. Furthermore, M1 macrophages lacking DGAT are susceptible to inflammation induced by palmitate ^[61]^. These results are supported by experiments that showed STAT6 and PPARδ/γ induce M2 macrophage polarization and prevented lipid-induced inflammation and cell death ^[62]^. Overall, macrophage phenotype and function in vivo are linked to pathways that regulate fatty acid uptake (CD36), storage (DGAT), or FAO and oxidative phosphorylation (CPT1, TFAM).

## 4. Chronic NLRP3 inflammasome activation is tied with FAO during aging

Macrophages rely on inflammasomes, crucial multiprotein complexes, to respond to danger signals, tissue damage, and cellular stress ^[63,64]^. The NLRP3 inflammasome, a well-described canonical inflammasome, forms an oligomer upon sensing various stimuli in a two-step process. Signal 1, considered priming by pathogen-associated molecular patterns (PAMPs), upregulates pro-inflammatory molecules and cascades, particularly NF-κB transcriptional activity. One trigger is bacteria or microbiome components, which is derived from the aging leaky gut or advanced glycation end products (AGEs) ^[65]^. Signal 2 is triggered by damage-associated molecular patterns (DAMPs), and results in the activation of the NLRP3 protein, and its oligomerization with adaptor proteins. This leads to the auto-catalytic cleavage of caspase-1 which processes pro-interleukins (pro-IL-1β and pro-IL-18) into their active forms (IL-1β and IL-18). Moreover, caspase-1 catalyzes the cleavage of gasdermin D thereby facilitating the release of active interleukins (IL-1β and IL-18), and initiating pyroptosis, an inflammatory form of cell death. DAMPs include components of cellular debris, lipid components, or extracellular proteins that increase during tissue damage ^[66,67]^. While this pathway is required for recognizing cellular stress and tissue damage, chronic stimulation of the NLRP3 inflammasome leads to positive feedback and becomes the basis of inflammaging ^[68]^. Macrophages from old individuals exhibit an inflammatory phenotype with increased signaling through pattern-recognition receptors, and activation of the NLRP3 inflammasome ^[69–72]^. Here, we discuss the evidence that links a decline in FAO with increased inflammatory profile of macrophages, specifically focusing on the role of the NLRP3 inflammasome.

Both signal 1 and signal 2 in the activation of the NLRP3 inflammasome result in enhanced glycolytic activity ^[73]^. IL-1β-dependent upregulation of 6-phosphofructo-2-kinase/fructose-2,6-biphosphatase 3 (PFKFB3) results in to reinforced inflammatory phenotype, establishing a positive feedback loop. PFKFB3 metabolizes fructose-2,6-bisphosphate, and promotes M1 polarization ^[74]^. Macrophages exposed to DAMPs, such as amyloid-β (a fibrillar protein associated with the formation of plaques in Alzheimer's disease), induce glycolytic processes through an IL-1β-dependent activation of PFKFB3 ^[75]^. Moreover, fermentation of glucose products into lactic acid can trigger NLRP3 inflammasome activation and result in heightened enzymatic activity of PFKFB3 ^[38]^. In aged, sedentary mice, microglia exhibit a glycolytic metabolic profile characterized by increased PFKFB3 expression, reduced phagocytic activity, as well as, elevated cellular senescence with increased levels of β-galactosidase activity and p16INK4A ^[76]^. These results are consistent with a heightened inflammatory profile, increased glycolytic activity that leads to cellular senescence, a cell state associated with an inability to proliferate, but resistance to apoptosis. Monocytes from older individuals have differential methylation that can lead to increased chromatin accessibility of pro-inflammatory cytokines ^[77]^. These cell-intrinsic methylation changes could explain the propensity for inflammatory cytokine production and glycolytic metabolism that supports a downregulation of FAO.

Histone deacetylase 3 (HDAC3) that regulates transcriptional activity also promotes the NLRP3 inflammasome activation through the direct inhibition of FAO-related transcription. HDAC3 inhibits FAO and FAO-mediated oxidative phosphorylation in macrophages, providing a molecular brake on M2 polarization ^[78]^. In macrophages, HDAC3 amplifies the NLRP3 inflammasome activation by impairing FAO and increasing ROS secretion in the mitochondria. This regulatory mechanism is achieved through the deacetylation of hydroxyacyl-CoA dehydrogenase trifunctional multienzyme complex subunit alpha ^[79]^. Furthermore, HDAC3 promotes chronic inflammation and LPS-induced caspase-1 activation in M1 macrophages ^[80]^. HDAC3 may serve as a specific molecular link between epigenetic changes, reduced FAO and increased activation of the NLRP3 inflammasome.

There are already several lines of evidence indicating that HDAC3 may be important for age-related pathology as a result of its deacetylation activity on lipid metabolism genes. In intestinal enterocytes and adipocytes, HDAC3 promotes diet-induced obesity ^[81]^. Targeted suppression of class I HDACs enhance the oxidative capabilities of white adipose tissue and promote the development of the browning phenotype ^[82]^. Moreover, when *Hdac3* is ablated from adipose tissue, the acetylation levels of *Ucp1* and *Ppara* gene enhancers are elevated ^[83]^. Bochkis et al ^[21]^ discovered that the repression of nuclear receptors, including PPAR-α, PPAR-γ, and liver X receptor alpha (LXR-α), is reversed in livers from older individuals. This derepression activates genes associated with lipid synthesis and storage, mainly from decreased HDAC3 activity on specific regions associated with PPARα targets, but increased forkhead box protein A2 (FOXA2) binding. Ultimately, the reciprocal binding between HDAC3 and FOXA2 at PPAR-α, PPAR-γ, and LXR-α target sites leads to gene regulation associated with fatty liver development during aging. These results are consistent with increased deacetylation activity that supports impaired lipid metabolism in metabolic disease and aging, but whether similar genes are targeted in macrophages and monocytes remains to be explored.

It is important to note that FAO is also linked with activation of the NLRP3 inflammasome ^[84]^. Palmitate-BSA complex increases caspase-1 activation, IL-1β cleavage, and IL-1β/IL-18 secretion in wildtype BMDMs but not *Nox4* knockout BMDMs in response to nigericin and ATP. Deficiency of NADPH oxidase 4 (NOX4), which generates mitochondrial ROS species H_2_O_2_, reduces activation of the NLRP3 inflammasome, lipid uptake, oxygen consumption, and acetyl-CoA production. They showed these results depend on CPT1a activity, using etomoxir that promotes FAO ^[84]^. However, the concentration of the etomoxir inhibitor used in this study (200 μM) was very high. Whether this high concentration could indirectly affect NLRP3 activation through the off-target depletion of cellular CoA levels, as previously mentioned, is unclear ^[48]^. In contrast, a separate report details the finding that NOX4 also functions as an anti-inflammatory factor which promotes M2 polarization, by reducing NFκB activity and the NLRP3 inflammasome activation ^[85]^. The paradoxical functions of NOX4 may be explained by the complexities of redox signaling that can lead to additional intracellular signaling supporting tissue remodeling during injury ^[86]^.

During aging, catecholamine-stimulated lipolysis declines in an NLRP3-dependent manner ^[87]^. Adipose tissue macrophages around sympathetic nerves in 24-month-old mice upregulate catecholamine-degradation genes. This leads to reduced availability of NE that suppresses lipolytic activity in adipose tissue, impairing the breakdown of stored triglyceride and resulting in a decline in FAO ^[87]^. It is important to note that macrophage NE signaling contrasts from adipocyte NE signaling. NE-induced stimulation through the β2-adrenergic receptor leads to increased triglyceride storage as opposed to triglyceride breakdown. These results require *Dgat1* and hypoxia-inducible lipid droplet-associated (*Hilpda*) genes ^[88]^. In LPS activation of macrophages, there is also triglyceride accumulation through the upregulation of HILPDA protein, which in turn downregulates ATGL, leading to decreased triglyceride hydrolysis and lipolysis ^[89]^, in complete contrast with the lipolytic pathway that LPS activates in adipocytes via TLR signaling which activates lipolysis ^[90]^. Macrophages lacking HILPDA show decreased intracellular lipid levels and an increased accumulation of fluorescently labeled fatty acids, indicating that HILPDA restricts the availability of fatty acid pools for FAO ^[91]^. Generally, the increasing sequestration of NE by aging inflammatory adipose macrophages, coupled with heightened lipid content and the suppression of lipolysis in adipocytes, all play a part in the development of metabolic dysfunction in older individuals.

In summary, the continued activation of the NLRP3 inflammasome during aging acts to hinder FAO and fosters the onset of inflammaging. However, further understanding of how NLRP3-related factors such as HDAC3 and NOX4 converge to regulate chronic inflammation and FAO in aging myeloid cells is needed.

## 5. Reduced PPAR-α signaling and dysregulated FAO during aging

Monocytes contribute to chronic low-grade inflammation during aging ^[92,93]^. Several lines of publications identify a decrease in PPARα that reduces FAO during aging. PPAR-α is a nuclear receptor essential for promoting the expression of enzymes promoting FAO ^[94]^. PPAR-α forms a heterodimer with the retinoid X receptor to bind to the promoter regions of target FAO genes and initiate their transcription ^[95]^. Wang et al showed that monocytes from mice and humans displayed impaired FAO capacity, evidenced by the accumulation of lipid droplets, but decreased PPARα expression, and ATP ^[22]^. The authors also showed that aged monocytes had significantly reduced fatty acid uptake/transport, peroxisomal import/transport, and FAO gene transcription relative to monocytes from young mice ^[22]^. These changes could be reversed by activation of PPAR-α using fenofibrate, along with caloric restriction, which activated FAO pathways. Downregulated PPAR-α is correlated with increased lipid droplets, and increased tumor necrosis factor-alpha (TNF-α) levels. Neutralization of TNF-α reverses the aged environment of monocytes through upregulated PPAR-α, also increased FAO through activated AMPK ^[22]^. Although this study focused on blood-derived monocytes, these dysfunctional lipid-laden monocytes are likely recruited by chemokines to numerous tissues, where they differentiate into tissue-resident macrophages. Both fenofibrate and TNF-α neutralization aid in the reversal of inflammaging by promoting the reduction of pro-inflammatory cytokines such as IL-6 ^[22]^. Consistently, other studies support the role of TNF-α, and IL-1β, in inhibiting PPAR-α transcription through the NF-κB pathway in Kupffer cells ^[32]^.

In line with this, in rat livers, aging results in decreased PPAR-α protein levels, as well as decreased levels of the downstream FAO targets *Acox1*, *Cpt1*, and *Cyp4a1* ^[23]^. Chung et al ^[19]^ showed that the NLRP3 inflammasome activation and IL-1β production were significantly increased in aged rat livers and positively correlates with Kupffer cell number. IL-1β treatment reduced the expression of PPAR-α in hepatocytes in a time-dependent manner. Furthermore, in kidneys, the expression of PPAR-α and FAO-related proteins were decreased as lipids accumulated in the renal tubular epithelial region in old rats. Authors pinpointed miR-21 as a microRNA candidate responsible for a substantial decrease in PPAR-α during aging, thereby inhibiting FAO. Additionally, the anti-inflammatory adipokine, adipolin, acts through PPARα to reduce inflammasome activation in the kidney ^[30]^. Other papers further consolidate how IL-1β inhibits PPAR-α activity. Li et al found quite interesting results related to metabolic programming and inflammation utilizing human monocytes (THP1 cells) ^[31]^. In contrast to typical M1 macrophages, which exhibit low oxidative phosphorylation and high glycolysis rates, THP1 cells exposed to hepatitis B virus (HBV) have higher oxidative phosphorylation and lower glycolytic rates, which extends to HPV-activated Kupffer cells. The heightened oxidative phosphorylation in these cells attenuates IL-1β, which limits the ability to maintain a strong antiviral response. Importantly, these cells are capable of suppressing HBV replication via IL-1β production. The authors utilized electrophoretic mobility shift assays to assess binding of PPAR-α to the enhancer region ENI of HBV. IL-1β reduced the binding of PPAR-α to the enhancer region of HBV without affecting the binding of other nuclear receptors. FAO was not analyzed in these studies, however these data support a role for IL-1β, in reducing PPAR-α activity ^[31]^. The extent of the involvement of IL1β, miR-21, other microRNAs, and PPAR-α-ability to interact or bind with distinct enhancer regions in aging macrophages, monocytes, and microglia remains unclear.

Inflammation, triggered by the receptor for advanced glycation end products (RAGE) through the activation of NF-κB, can contribute to metabolic imbalances, including reduced PPAR-α expression ^[96]^. Initially known for its role in binding to AGEs, RAGE interacts with a group of molecules formed through nonenzymatic reactions involving sugars and proteins or lipids. Activated macrophages are a source of AGEs ^[97]^. The buildup of these AGEs in tissues, particularly in conditions like diabetes and during the aging process, is associated with vascular damage. RAGE exhibits versatility by binding to various other ligands, such as HMGB1, S100 proteins, β-amyloid peptides, MAC-1, and LPS, resulting in a diverse array of cellular responses. As age progresses, RAGE expression increases and consequently decreases PPAR-α expression ^[24]^. Reducing RAGE levels curbs macrophage presence and reduces pro-inflammatory cytokines but raises PPAR-α levels in older mice. On the other hand, overexpression of RAGE in middle-aged mice leads to increased infiltration of macrophages, elevated pro-inflammatory cytokines, and decreased PPAR-α. These findings highlight the importance of studying the relationship between inflammatory and metabolic pathways as aging progresses.

Aging-associated diseases, such as Alzheimer’s disease, reveal insight into FAO dysregulation in microglia that is linked to PPAR-α transcriptional activity. Burns et al ^[26]^ demonstrated an increase in unique auto-fluorescent (AF^+^) microglial populations in the healthy brain during aging. In mice, and nonhuman primates, AF^+^ microglia increase in size and obtain more lysosomal storage bodies. AF^+^ microglia have a highly catabolic metabolism, relying heavily on FAO, which increases concentration of ROS during aging. The accumulation of lysosomes in AF^+^ microglia reduces their autophagic function. The authors further show that AF^+^ microglia from 24-month-old mice exhibit a 2-fold increase of ROS compared to AF^+^ microglia from young counterparts, and a 2.4-fold increase of ROS compared to AF^-^ microglia. Predicted proteomic pathway analysis indicates activation of NFKB1A and PPARA in AF^+^ microglia, as well as CDKN2A, which is also present in age-dependent cellular senescence in microglia ^[98]^. In human microglial cells, administration of PPAR-α agonists enhances autophagy ^[25]^. Consistent with this, treatment with these agonists in Alzheimer’s disease mouse model reduces soluble and insoluble amyloid-β, memory deficits and anxiety with enhanced microglial recruitment and autophagosome biogenesis ^[25]^. This presents a complex model, where upregulated FAO pathways may be an attempt to support elevated levels of lysosomal autophagy, but over time results in mitochondrial dysfunction and increased ROS levels. Moreover, these results suggest that the dysfunction of a single enzyme in the FAO pathway does not support cellular aging, but rather it is the overall disruption of energy homeostasis.

In summary, activation of the NLRP3 inflammasome and increased IL-1β/TNF-α in aging-related macrophages and monocytes lead to disruption of FAO in these cells and tissues. Additionally, accumulation of RAGE with aging contributes to metabolic imbalance with decreased PPAR-α. These findings collectively suggest that chronic inflammation reduces PPAR-α levels. Further investigations into age-related alterations in microRNAs, exosomes, chromatin accessibility, and mitochondrial stress, in macrophages, monocytes, and microglia, which may lead to chronic inflammation, may help identify specific sequences or combinations of events that contribute to cellular and organ aging.

## 6. Aging, AMPK signaling, and dysregulated FAO

AMPK is a regulator of cellular energy balance which becomes activated when cellular energy levels are low, but it is activity is reduced in older individuals ^[99]^. The specific reasons for age-related reduced AMPK activity are not fully understood; however, decreased autophagy, increased cellular stress, increased inflammation are all downstream results. Under healthy conditions, it is activation restores energy homeostasis by promoting ATP-producing catabolic pathways and inhibiting energy-consuming pathways. Directly related to lipid metabolism, AMPK-α phosphorylates ACC at Ser79, resulting in the suppression of its enzymatic function. Inactivation of ACC leads to a decrease in malonyl-CoA levels and reduces fatty acid synthesis ^[100]^. The inhibition of CPT1 is relieved through the inhibition of ACC, enabling increased transport of fatty acids into the mitochondria for oxidation. The AMPK signaling pathway also plays a pivotal role in reducing inflammation and regulating myeloid cell differentiation and polarization ^[101,102]^. These results are in line with a role for reduced AMPK activity supporting dysfunctional myeloid cell differentiation and polarization in older individuals.

The need for AMPK signaling in monocyte-to-macrophage differentiation is described in experiments that examine the ability of macrophage colony-stimulating factor, a critical growth factor for macrophage differentiation, to activate AMPK and FAO ^[103]^. Deficiency of the catalytic subunit of AMPK impairs autophagy-mediated monocyte differentiation and promotes apoptosis of those cells ^[103]^. Conditional deletion of *Cpt1a* or using etomoxir, in monocytes also results in impaired monocyte-to-macrophage differentiation, and reduced inflammation ^[104]^. Like macrophages, LPS-exposed monocytes undergo a metabolic shift towards aerobic glycolysis. In sites of heightened inflammation, glucose levels are often decreased. To evaluate whether monocytes are capable of performing major functions negating the Warburg effect, Raulien et al ^[105]^ conducted an interesting study. Monocytes deprived of glucose exhibit lower levels of ROS, due to activated AMPK signaling, as compared to their glycolytic LPS-treated counterparts. Furthermore, glucose-deprived monocytes still secrete pro-inflammatory cytokines and perform phagocytosis at sites of inflammation, due to a respiratory burst and an increase in oxidative phosphorylation. This metabolic flexibility was also examined by Minhas et al ^[27]^, where they found that young monocyte-derived macrophages (MDMs) can offset the effects of glycogen synthase activity and PGE_2_-induced glycogen synthesis by utilizing alternative carbon substrates, including glucose, pyruvate, lactate, and glutamine. Conversely, aged MDMs from old mice lack this metabolic plasticity as they are solely dependent on glucose/glycogen as their principal carbon reservoir, which is insufficient for ATP synthesis. They linked this to an age-associated increase in PGE_2_ levels, which exerts a pro-inflammatory effect signaling through its EP2 receptor leads to an impairment in metabolic plasticity that could also involve AMPK signaling ^[27,106]^. AMPK is also linked to macrophage polarization. In macrophages, transfection of a constitutively active form of AMPK-α1 leads to increased production of IL-10, diminished LPS-induced pro-inflammation, through reducing the degradation of IκB-α, and pro-inflammatory gene expression ^[100]^. AMPK is known to increase cellular NAD^+^ levels and SIRT1 activity resulting in the deacetylation of SIRT1 targets including PPAR-α, and NFkB, among others ^[107]^. In microglia in cell cultures or in mouse models, amyloid-β induces spleen tyrosine kinase activation leading to reduced AMPK signaling, but increased AKT signaling ^[108]^. Furthermore, the inactivation of AMPK leads to fragmentation of mitochondria, an increase in the production of ROS, and activation of the NLRP3 inflammasome ^[28,108]^. These results indicate that reduced AMPK activity acutely alters cellular signaling and mitochondrial function, but chronic conditions of reduction would support permanent transcriptional alterations. Cell nonautonomous effects of reduced AMPK in macrophages and microglia, include reduced insulin sensitivity in adipocytes ^[109]^, and an accumulation of amyloid-β due to reduced CD36 expression ^[110,111]^. In summary, macrophage differentiation and cytokine production depends on AMPK and FAO and relies on signaling between EP2, SIRT1, AKT and activation of the inflammasome. AMPK-induced metabolic flexibility allows them to transition from glycolysis to FAO; where some data suggests that metabolic flexibility diminishes with aging ^[27]^. However, aged classical monocytes from humans maintain effector and metabolic function during glucose deprivation ^[112]^, suggesting that there may be species or cell-specific differences.

Two well-studied methods to activate AMPK signaling in older individuals include calorie restriction or the use of metformin ^[113,114]^. Caloric restriction increases AMPK signaling, FAO activity and reduces inflammation in monocytes ^[22]^. Those results are consistent with a large body of literature that provides evidence for calorie restriction in reducing ROS, mitochondrial dysfunction and inflammation, while improving cellular metabolism and overall lifespan ^[114]^. Metformin, primarily prescribed for managing elevated blood sugar in individuals with diabetes mellitus, holds promise as a potential antiaging medication ^[115,116]^. Recent discoveries have revealed its action as a SIRT1/AMPK agonist, enhancing FAO and extending both lifespan and health span ^[116–118]^. Metformin also increases Dicer1, a miRNA-processing enzyme that could explain a reduction in inflammatory or senescent phenotype ^[119]^. miR-146a is increased in senescent fibroblasts, and its overexpression reduces NAD metabolism and SIRT activity ^[29]^. Inhibition of miR146a accelerates the senescent phenotype through the same pathway. Metformin and AMPK activator 5-aminoimidazole-4-carboxamide-1-β-d-ribofuranoside, through activating FAO and AMPK, reduce miR-146a production and reduce inhibits NF-κB via increased IκB kinase activity. Additional research into the effects of environmental changes (like calorie restriction or exercise) or pharmaceutics (like metformin) on macrophage-derived AMPK activity is still needed to understand specific molecular links.

## 7. Final Remarks and conclusions

**Figure 1.**
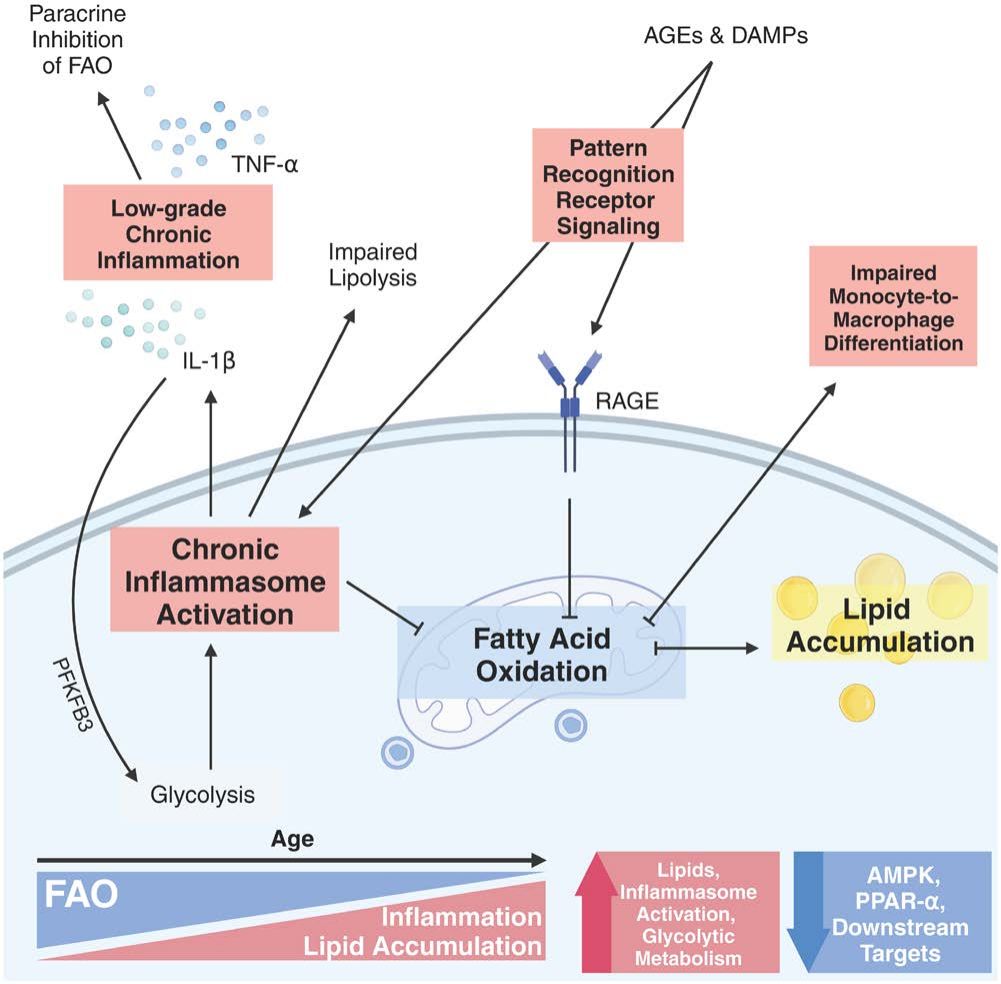
**Mechanism of fatty acid oxidation dysregulation during aging.** This schematic gives a broad overview of the changes seen in macrophages and monocytes from older individuals. During aging, chronic stimulation by PAMPs and DAMPs causes the activation of pattern-recognition receptor pathways, like RAGE, TLRs, and the NLRP3 inflammasome. This increases pro-inflammatory cytokines like TNF-α and IL-1β and promotes the polarization of M1 macrophages that exhibit a pro-inflammatory phenotype. M1 macrophages also exhibit a robust shift towards glycolysis, which is supported by PFKFB3. These cytokines downregulate PPARα and FAO activation. Macrophages during aging also exhibit a downregulation of AMPK signaling, increased triglyceride synthesis, an accumulation of lipid droplets. Decreases in AMPK signaling leads to impaired monocyte-to-macrophage differentiation, reduced FAO, accumulating ROS, and amplified activation of the NLRP3 inflammasome. Arrows pointing directly outwards from a central point represent positive/feed-forward processes, whereas arrows branching off from an inhibition line denote processes that develop as a result of downregulated pathway (eg, FAO). The figure was created with Biorender.com. AMPK, 5’ AMP-activated protein kinase; FAO, fatty acid oxidation; IL-1β, interleukin-1 beta; NLRP3, NLR family pyrin domain containing 3; PFKFB3, 6-phosphofructo-2-kinase/fructose-2,6-biphosphatase 3; PPAR-α, Peroxisome proliferator-activated receptor alpha; RAGE, receptor for advanced glycation end products; ROS, reactive oxygen species; TNF-α, tumor necrosis factor-alpha.

Low-grade chronic inflammation and immune activation increase within metabolic organs during aging. This generates an environment of elevated secreted factors that promote inflammatory signaling pathways, including RAGE, NLRP3 inflammasome, and NFκB, that initiate metabolic dysfunction via downregulation of PPAR-α, SIRT1, and AMPK. A prominent metabolic change within macrophages includes downregulation of FAO and reliance on glycolysis to generate energy. This is a complex feed-forward cycle, where dysregulated and altered FAO in macrophages and monocytes also has significant implications for promoting chronic inflammation (Figure 1). However, further research is needed to explore the heterogeneity of macrophage and monocyte populations in vivo and uncover FAO capacity in different tissues, and ages. Several lines of work indicate that therapeutically targeting metabolic pathways, like FAO, could reduce their inflammatory profile. Under the diversity of human diseases, it would be important to understand whether therapeutic changes are temporary or permanent. Understanding these distinctions may enable the modulation of macrophage and monocyte activities for therapeutic interventions. In summary, future studies should consider age as a variable when examining metabolic pathways.

## Author contributions

V.K. wrote the manuscript, generated the conceptual ideas, and made the figure. I.H.J. and C.D.C. provided scientific feedback, and comments on the manuscript.

## Conflicts of interest

The authors declare that there are no conflicts of interest.

## Funding

This work was supported by National Institute of Health grant R00 AG058800 and R01 AG AG079913 (C.D.C.), the McKnight Land-Grant Professorship (C.D.C.), the Glenn Foundation for Medical Research/AFAR Grants for Junior Faculty (C.D.C.), and the Medical Discovery Team on the Biology of Aging.

## Acknowledgments

Thank you to Stephanie Cholensky for edits of the drafts. Biorender was used to generate schematics under the Agreement #’s: HU26213HPY.
